# Targeted antitumour therapy – future perspectives

**DOI:** 10.1038/sj.bjc.6602606

**Published:** 2005-05-31

**Authors:** M Ranson, G Jayson

**Affiliations:** 1 Department of Medical Oncology, University of Manchester, Christie Hospital NHS Trust, Wilmslow Road, Manchester M20 4BX, UK

**Keywords:** targeted therapy, antitumour, clinical trial design, biomarker

## Abstract

The advent of targeted therapy presents an unprecedented opportunity for advances in the treatment of cancer. A key challenge will be to translate the undoubted promise of targeted agents into tangible clinical benefits. Achieving this goal is likely to be dependent upon a number of factors. These include continued research to improve our understanding of the heterogeneity and complexity of the tumour microenvironment; refinement of clinical trial design to incorporate nontraditional end points such as the optimum biological dose and health-related quality of life; and the use of technological advancements in proteomics, genomics and biomarker development to better predict tumour types and patient subsets that may be particularly responsive to treatment, as well as enable a more accurate assessment of drug effect at the molecular level. In summary, the future success of targeted agents will require an integrated multidisciplinary approach involving all stakeholders.

The development of targeted agents holds considerable promise for cancer treatment but progress to date has not been easy. Many of the difficulties encountered in the development of targeted anticancer agents can be explained by an incomplete understanding of human tumour biology, limited understanding of the drug target and problems with patient selection. The development of targeted anticancer agents has required novel trial designs as well as the investigation of new pharmacodynamic and surrogate trial end points. The future success of targeted therapy will involve initiatives to identify the most promising targets, closer integration of preclinical and clinical data, a greater use of genomic and proteomic techniques, and further refinement of trial design.

## SELECTING TARGETED AGENTS FOR TARGETED PATIENT POPULATIONS

Most cancers are driven by and dependent upon multiple aberrant signalling pathways, and thus ‘single-hit’ therapy may not represent an optimal approach in many clinical situations. It is evident that there is scope for significant redundancy in cell signalling and selecting a single target within a heterogeneous tumour type may yield clinically disappointing results. In tumours with a relatively narrow range of critical genetic defects (e.g., acute promyelocytic leukaemia and chronic phase chronic myeloid leukaemia (CML)), the ability to develop successful targeted agents with striking activity has been more easily accomplished than in more complex and heterogeneous tumour types (e.g., breast cancer, and non-small-cell lung cancer (NSCLC)). Most clinically apparent tumours contain multiple genetic defects with incompletely defined phenotypic consequences. The declining therapeutic impact of imatinib as one proceeds in Philadelphia-positive CML from chronic-phase disease to accelerated phase, and finally to blast crisis, illustrates the impact of increasing genetic alterations in conferring drug resistance to a targeted agent (Gorre *et al*, 2001; von Bubnoff *et al*, 2002). For successful targeted drug development we ideally need to be able to identify targets that provide a critical transforming signal to the tumour as distinct from targets where expression is not linked with signal-dependency. We must also acknowledge that targeted drugs may have the most marked effects on only a subset of tumour cells and may be incapable of inhibiting quiescent and perhaps non-‘target-addicted’ tumour stem cell populations. Targeted drugs may therefore be able to provide tumour control rather than cure.

The clinical evaluation of targeted agents has provided some refinement in the classification of complex tumours, and this trend is likely to gather pace as tumours become defined by molecular features rather than according to light microscopic classification. The emergence of trastuzumab as a useful agent in a subset of breast cancer patients is one example of the impact of targeted treatment in refining tumour classification (Vogel *et al*, 2002). Likewise, the finding that a small subset of NSCLC patients who have gain-of-function epidermal growth factor receptor (EGFR) mutations and response rates to gefitinib and erlotinib of over 80% must be viewed as a major advance in our understanding of lung cancer biology (Lynch *et al*, 2004; Paez *et al*, 2004; Mitsudomi *et al*, 2005; Tokumo *et al*, 2005). The future clinical evaluation of targeted agents will shed new light on tumour pathogenesis, provide better understanding of tumour biology and result in changes to the way that tumours are classified.

For trastuzumab (Herceptin®) and imatinib (Glivec®), a limited number of molecular features can be used to define a suitable target patient population, but it seems unlikely that all patients who could derive clinical benefit from targeted drugs will be identified by determination of a single molecular feature. We know also that tumour architecture is heterogeneous and may significantly affect the intratumoural pharmacokinetics of a drug (Jayson *et al*, 2002). Thus, a drug that is effective at the molecular level may not necessarily achieve clinically useful doses in the tumour.

Complex tumour types may be more susceptible to the use of multiple-targeted agents or to drugs that target generic, fundamental tumour processes such as angiogenesis, apoptosis and cellular proliferation. For example, small molecule tyrosine kinase inhibitors (TKIs) such as ZD6474 (inhibitor of vascular endothelial growth factor receptor (VEGFR) and EGFR) (Wedge *et al*, 2002; Ciardiello *et al*, 2003), SU11248 (inhibitor of VEGFR, platelet-derived growth factor receptor (PDGFR), c-Kit, colony-stimulating factor receptor-1 (CSFR-1) and FLT3-R) and vatalanib (PTK787/ZK222584; inhibitor of VEGFR and PDGFR) (Abrams *et al*, 2003; Thomas *et al*, 2003) show activity against more than one receptor and are inhibitory in a wide range of preclinical models.

Since there are no predefined predictive rules, a major future challenge must be to identify critical molecular events that drive tumour growth, survival and metastasis. Important determinants of tumour behaviour are likely to be DNA mutational or rearrangement events, which produce oncogenes with dominant gain of function or tumour suppressor genes with recessive loss of function.

## CLINICAL TRIAL DESIGN

The traditional phase I trial design used for cytotoxic drugs has been recognised as having limitations for targeted agents. Instead of the maximum tolerated dose, definition of the optimum biological dose became a central theme in phase I studies (Gelmon *et al*, 1999; Korn *et al*, 2001). However, a retrospective review of 60 phase I trials of targeted agents reported mainly between 1999 and 2002 revealed that the majority of studies had used toxicity as the sole determinant for dose selection in phase II studies. Nontraditional end points such as measurements of drug effect or functional imaging have not been routinely incorporated into phase I trial design and have rarely formed the basis for future dose selection or trial design (Parulekar and Eisenhauer, 2004).

Proof-of-principle studies to measure drug effect on target are highly desirable during early drug development, but are difficult to perform. Although tumour represents the tissue of primary interest, tumour sampling can be obtained in only a limited proportion of patients and at a small number of time points. Trials with mandatory biopsies have greater ethical and administrative complexity and the net result is often slower patient recruitment and recruitment of a narrow range of tumour types. Preclinical models are needed that define an appropriate degree of target inhibition for clinical studies, but observations of target inhibition still need to be clinically validated. Surrogate tissues (peripheral blood mononuclear cells, skin, hair follicles) may be useful in that they can be more readily collected than tumour biopsies, but the pharmacodynamic relationship between surrogate and tumour cells needs to be understood and defined. The incorporation of surrogate and tumour pharmacodynamic markers into clinical trials has also highlighted the fact that assay validation, reliability and quality assurance need attention if results are to be interpretable (Colburn, 2003; Cummings *et al*, 2004).

Once an agent has been found to have acceptable toxicity in phase I (ideally with proof-of-principle that it has achieved inhibition of the putative biological target), it is necessary to demonstrate that it has clinical activity in phase II and III trials. Patient selection for such trials is often problematic, particularly if there is no established link between the chosen target and a ‘diagnostic signature’. Translational research has established a significant correlation between genomic amplification and high expression of HER2/neu in advanced breast cancer, and response and clinical benefit from trastuzumab (Vogel *et al*, 2002). However, the predictive capacity of this observation has its limitations, since despite the development of validated molecular diagnostics for HER2, only a minority of patients with HER2/neu-positive tumours will actually respond to trastuzumab monotherapy. However, the relatively high response rates in phase II/III trials of imatinib in metastatic gastrointestinal stromal tumours (GIST) support the intrinsic power of a science-driven approach to patient selection (van Oosterom *et al*, 2001; Demetri *et al*, 2002). Imatinib is an inhibitor of c-Kit receptor tyrosine kinase, and constitutive activation of this receptor, resulting from gain-of-function mutations in the c-Kit gene, had been hypothesised to be critical in GIST (Rubin *et al*, 2001). The situation for EGFR TKIs has however been more challenging. In this case, the target receptor was found to be widely expressed in epithelial tumours and strong preclinical data supported a role for EGFR in epithelial tumour biology. Nevertheless, the relationship between EGFR expression, target inhibition and tumour dependency on EGFR was unclear, and it was clinical observations that propelled a breakthrough in basic science. Responses to gefitinib in advanced NSCLC were encountered in a minority of patients who more frequently had adenocarcinoma, were non- or ex-smokers, and were more likely to be female or Japanese (Fukuoka *et al*, 2003; Kaneda *et al*, 2004; Miller *et al*, 2004). Molecular characterisation of responding patients identified a novel subset of NSCLC patients with gain-of-function mutations in the ATP-binding domain of the receptor (Lynch *et al*, 2004; Paez *et al*, 2004; Mitsudomi *et al*, 2005; Tokumo *et al*, 2005). Had this biology been known in advance, patient selection based upon this criterion could have yielded a response rate to gefitinib and erlotinib of over 80% in phase II trials in this subset of NSCLC. Analysis of randomised clinical data with erlotinib suggests that this select cohort of mutation-positive patients does not fully explain the survival differences between erlotinib and best supportive care in relapsed NSCLC (Shepherd *et al*, 2004), and it is sobering to reflect that this observation would be precluded by ‘*a priori*’ selection of patients based on EGFR mutation status. Similarly, the careful evaluation of imatinib-resistant patients has fuelled new understanding of CML biology and facilitated the development of second-generation lead compounds (Ross and Hughes, 2004). While there is an overwhelming case for strong science-driven trials, the ability of trials to drive basic science should not be underestimated.

It is unlikely that all patients who will experience clinical benefit will be identified by ‘single gene’ analysis or a single molecular marker because of the complexity of most tumour phenotypes. Genomic and proteomic profiling to examine a larger number of genes and proteins can elucidate more complex molecular signatures, and a number of such profiles have been defined, which predict survival or treatment response (Van't Veer *et al*, 2003; Yanagisawa *et al*, 2003; Carr *et al*, 2004). These technologies can also be applied to targeted therapies, although greater efforts, including some degree of speculative translational research on clinical samples, will be required to develop this area rapidly.

The choice of response rate as a primary clinical end point in phase II trials of targeted drugs needs to be questioned. Tumour regression has remained the benchmark against which drugs are normally assessed in phase II despite the fact that time to progression may be a more relevant phase II end point for many targeted agents. In addition, some of these novel agents may take longer to stabilise disease than traditional cytotoxic agents, and we may need to use less stringent definitions of progressive disease so that patients can be exposed to an experimental drug for longer.

## IDENTIFYING AND VALIDATING MARKERS OF TARGET INHIBITION

Although most phase I trials of targeted drugs incorporate translational science, the same cannot be said of many phase II or III trials despite the fact that this arena is likely to be one of the most fruitful for translational science. The ability to perform analysis at DNA, RNA and protein levels and to correlate this with outcome in phase II and III trials should facilitate our ability to reclassify tumours and identify subpopulations of patients who gain from targeted therapeutics. In practice, most patients are willing to have additional investigative studies performed on their tumours in the hope that it may yield important information. It is important that all stakeholders involved in the clinical testing of new anticancer agents understand that the correlation of such data with clinical outcome may be critical for the optimal development and use of current and future targeted therapies. Thus, incorporating translational research into phase II and III trials needs to be recognised as an important future objective.

Dose delivery with cytotoxic drugs is normally heavily influenced by normal tissue toxicity (e.g., myelosuppression and mucositis). It seems a reasonable premise that dosing for targeted therapies should be driven by target effect rather than toxicity. While this does not remove the need to evaluate traditional clinical end points (toxicity, tumour response, time to progression and survival), there is increasing interest in identifying biomarkers of drug effect. With the relatively low toxicity of many targeted agents compared with conventional treatment strategies, the short-term administration of a targeted agent before elective surgery may be a useful method for identifying biomarkers that merit further clinical investigation.

## CONCLUSION

There are unprecedented opportunities to translate our understanding of tumour biology into more significant therapeutic gains. However, we must accept that our understanding of clinical human cancer is incomplete and that there are many obstacles to overcome. Identifying a molecular target that can be inhibited and then simply testing a promising lead compound in unselected patients may not be an effective strategy without determining target effect or conducting adequate translational research. As the numbers of patients treated with targeted agents increases, it will become possible to determine whether significant tumour dependency exists for a specific biological target, at least in a subset of patients. Indeed, phase III trials of bevacizumab have recently validated VEGF as a target in both CRC (Hurwitz *et al*, 2004). We should attempt to elucidate potential mechanisms whereby tumours circumvent this target dependency, as this may help us in patient selection and finding effective combination therapies. Initial clinical development should focus on proof-of-principle, a demonstration that the drug produces inhibition of the biological target at doses that are well tolerated, and that the consequences of target inhibition can be identified and measured using validated surrogates for clinical benefit. This will require the integration of pharmacokinetics, pharmacodynamics and possibly functional imaging into trials. Selection of a specific population of patients is desirable if adequate clinical or preclinical data exists. In the absence of data allowing patient enrichment, further preclinical work may be required or significant investment in gathering tumour and correlative material should be undertaken in phase I and II studies. Technologies such as genomics, proteomics and biomarker development represent promising approaches with which to probe for predictive markers. Everyone involved in developing novel targeted anticancer therapies should recognise that the most significant future advances are likely to emerge from the incorporation of such technologies into clinical trials. In short, the future success of targeted therapies will need a coordinated, multidisciplinary team approach.
